# A case of CRPC with multiple bladder invasions treated with EBRT followed by HDR-BT boost

**DOI:** 10.1259/bjrcr.20210039

**Published:** 2022-03-09

**Authors:** Yoshiaki Takagawa, Jun Itami

**Affiliations:** 1 Department of Radiation Oncology, Southern Tohoku Proton Therapy Center, Fukushima, Japan; 2 Department of Radiology, Tokyo Metropolitan Tama Medical Center, Tokyo, Japan; 3 Department of Radiation Oncology, National Cancer Center Hospital, Tokyo, Japan

## Abstract

We report a case of post-operative local recurrence of castration-resistant prostate cancer with multiple bulky bladder invasions treated using external beam radiotherapy (EBRT) followed by a high-dose-rate brachytherapy (HDR-BT) boost. The EBRT dose was 46 Gy delivered in 23 fractions with intensity-modulated radiotherapy to the entire pelvis. The HDR-BT dose was 15 Gy delivered in 1 fraction using ultrasound, CT, and MRI-guided brachytherapy with 18 interstitial needles. We achieved excellent local control of cancer in the prostate bed and multiple bulky bladder invasions. EBRT plus HDR-BT boost can allow higher doses to be delivered than EBRT alone for locally recurrent bulky prostate cancer following prostatectomy.

## Introduction

The optimal timing of radiotherapy after prostatectomy was unknown until the recent RADICALS-RT study showed that salvage radiotherapy is less toxic than adjuvant radiotherapy.^1^ The RAVES and GETUG-AFU 17 studies also reported a higher incidence of genitourinary (GU) toxicity in the adjuvant radiotherapy group.^2,3^ The National Comprehensive Cancer Network (NCCN) guidelines recommend adjuvant or salvage radiotherapy after prostatectomy at 64–72 Gy via external beam radiotherapy (EBRT) in standard fractions.^4^ However, the American Urological Association/American Society for Radiation Oncology guidelines for adjuvant and salvage radiotherapy after prostatectomy do not mention high-dose-rate brachytherapy (HDR-BT) in the context of salvage radiotherapy.^5^

Gross recurrent tumors in the prostate bed have occasionally been reported after prostatectomy. The NCCN guidelines state that biopsy-proven gross recurrence may require higher treatment doses. If a recurrent tumor is detected macroscopically on multiple imaging studies, EBRT plus HDR-BT boost may allow higher doses to be delivered than EBRT alone.

We report a case of post-operative local recurrence of castration-resistant prostate cancer (CRPC) with multiple bulky bladder invasions treated using EBRT followed by HDR-BT boost.

## Clinical presentation

A 69-year-old male was diagnosed with high-risk prostate cancer with a Gleason score of 9 (4 + 5) based on a prostate biopsy performed in 2006. The initial prostate-specific antigen (PSA) level was 9.8 ng ml^−1^, and the clinical stage was T3N0M0. Definitive, robot-assisted, radical prostatectomy revealed the pathological stage to be T3bN0M0 with a Gleason score of 9 (5 + 4). This primary surgery was performed at another hospital. Therefore, the details regarding pathological features (*e.g.* pR0 or pR1 resection) were unfortunately unavailable in our medical records. The post-operative PSA nadir was also unknown. 1 year after prostatectomy, his PSA level increased by 0.946 ng ml^−1^, and he was diagnosed with failure of prostatectomy. Salvage hormonal therapy (bicalutamide and leuprorelin acetate) was administered. After 4 years of hormonal therapy, the PSA level was <0.008 ng ml^−1^. However, he stopped hormonal therapy for 7 months, and his PSA level increased to 1.362 ng ml^−1^, following which, hormonal therapy was restarted. Nevertheless, the PSA level gradually increased from 2.974 ng ml^−1^ in 2015 to 4.833 ng ml^−1^ in 2016. Thereafter, he presented to our hospital (National Cancer Center Hospital) for treatment. He was diagnosed with CRPC in 2017.

**Figure 1.**
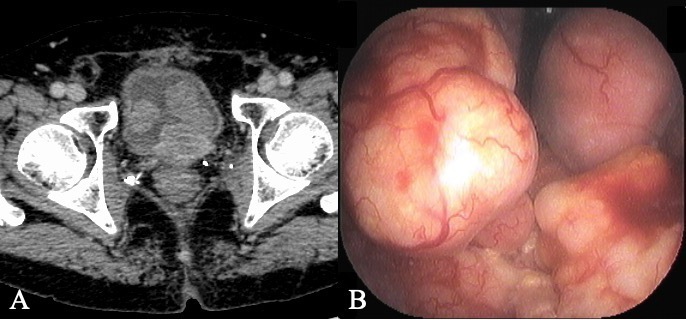
**(A**) CT shows tumor recurrence in the prostate bed with multiple bulky bladder invasions 1 year after enzalutamide administration. (**B**) Cystoscopic findings of the bladder tumor before salvage radiation therapy.

CT revealed local recurrence of the tumor in the prostate bed. Enzalutamide was administered, but the tumor continued to grow. Multiple bulky bladder invasions were visualized on CT after 1 year (Figure 1A). The patient complained of dysuria, hematuria, and urinary incontinence. He refused chemotherapy and was referred to the Department of Radiation Oncology for salvage radiotherapy. Restaging fluorodeoxyglucose-positron emission tomography (FDG-PET)/magnetic resonance imaging (MRI) showed no distant metastases. Cystoscopy and biopsy of the bladder tumor (Figure 1B) confirmed adenocarcinoma with a Gleason score of 9 (4 + 5).

## Treatment

**Figure 2.**
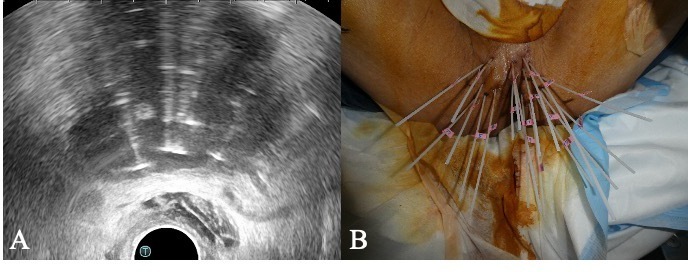
**(A**) Real-time TRUS image after interstitial needle implantation. (**B**) Photograph of HDR-BT after interstitial needle implantation. 18 needles percutaneously inserted into the target. HDR-BT, high-dose-rate brachytherapy; TRUS, transrectal-ultrasound.

**Figure 3.**
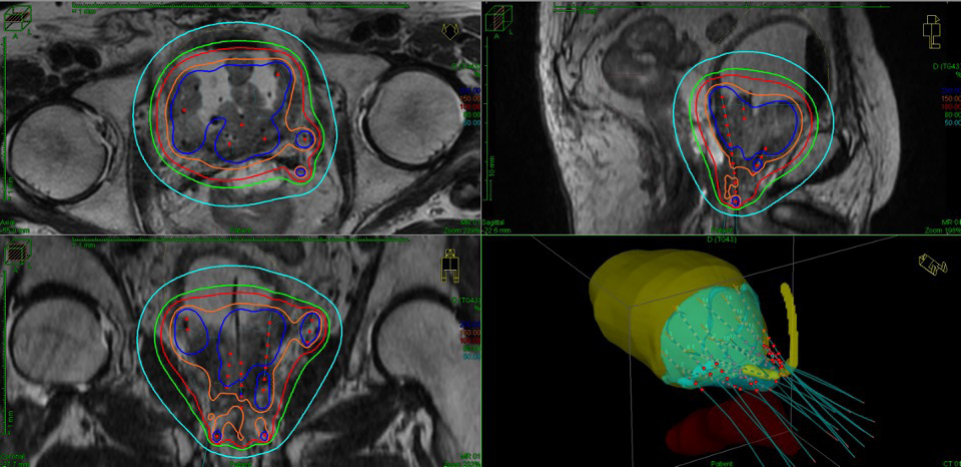
MRI image fused with planning CT after interstitial needle implantation. Applicator reconstruction and dose distribution of HDR-BT are demonstrated. 15 Gy (red line) was prescribed for 100% of the clinical target volume. HDR-BT, high-dose-rate brachytherapy.

EBRT alone was considered insufficient for controlling the recurrent tumors, given the extent of the disease. Thus, we decided to perform EBRT with intensity-modulated radiotherapy (IMRT) followed by an HDR-BT boost. Before EBRT, we inserted a hydrogel spacer (SpaceOAR^®^, Boston Scientific, Marlborough, MA) between the prostate bed and rectum to reduce the rectal dose. The maximum PSA level before radiotherapy was 31.723 ng ml^−1^. The EBRT dose was 46 Gy, delivered in 23 fractions to the entire pelvis. 6 days after the completion of EBRT, MRI showed that although there was slight shrinkage, the recurrent tumor remained bulky. 2 weeks after the completion of EBRT, HDR-BT was performed. The prescribed dose was 15 Gy in 1 fraction. For remote afterloading, we used microSelectron HDR-V3 with Oncentra Brachy (Elekta, Sweden) with Ir-192. We inserted 18 ProGuide Sharp Needles (Elekta, Sweden) with an outer diameter of 1.67 mm into the tumor under local anesthesia and transrectal ultrasound (TRUS) guidance (Figure 2). During needle implantation, we performed a CT scan to optimize the positions and depths of the needles. Second, after needle implantation, planning CT and MRI were performed. The acquired MRI were fused to the planning CT, and we contoured the target and organ-at-risks on the treatment planning system (Figure 3). If any needle was slightly shallower or deeper than planned on MRI, we modified its depth to achieve the position as per the planning CT. We contoured the tumor in the bladder neck and prostate bed to determine the clinical target volume (CTV), which was 58.35 cc. We applied the dose constraints of HDR monotherapy for prostate cancer according to our institutional protocol. Table 1 lists the dosimetric parameters and dose constraints. The total biological effective dose of EBRT plus HDR-BT was 272 Gy, assuming an α/β ratio of 1.5.

**Table 1.** Dosimetric parameters of HDR-BT

Dosimetric parameters		Dose constraints per institutional protocol
**CTV**		
Volume	58.35 cc	
D_90_	20 Gy	>15 Gy
V_100_	99.6%	>95%
V_150_	78.37%	
V_200_	50.07%	
**Urethra**		
D_max_	22.4 Gy	
V_110_	0.36 cc	<1 cc
**Rectum**		
D_max_	13.7 Gy	
D_1cc_	10.2 Gy	
D_2cc_	9.3 Gy	<10.5 Gy
**Bladder**		
V_125_	72.8 cc	<1 cc

Bladder V_125_, fractional volume of bladder receiving 125% of prescribed dose; CTV, clinical target volume; D_90_, minimal dose delivered to 90% of target volume; HDR-BT, high-dose-rate brachytherapy; Rectum D_max_, maximum point dose for rectal volume < 115%; Urethra D_max_, maximum point dose for urethral volume < 115%; Urethra V_110_, fractional volume of urethra receiving 110% of prescribed dose; V_n_ (_100_, _150_, _200_), fractional volume of the organ receiving n% of the prescribed dose; rectum D_1cc_ and D_2cc_, doses for most exposed 1 cc and 2 cc volumes of rectum.

**Figure 4.**
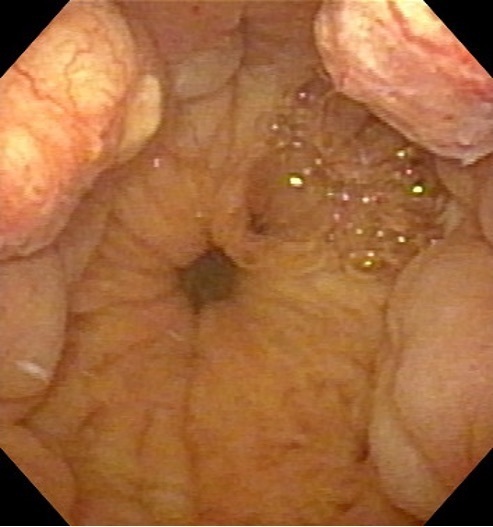
Cystoscopy shows good reduction in the multiple bladder invasions three months after HDR-BT. HDR-BT, high-dose-rate brachytherapy.

3 months after HDR-BT, the PSA level decreased to 6.970 ng ml^−1^, and cystoscopy showed a reduction in bladder invasion (Figure 4). Acute toxicities included Grade 1 dysuria and Grade 1 hematuria, which pre-existed and did not worsen after radiotherapy. However, 4 months after HDR-BT, the PSA level increased again, and multiple bone metastases were detected. The patient experienced numbness and weakness in his left hand. We administered palliative EBRT at a dose of 46 Gy in 23 fractions for vertebral metastasis. Thereafter, his symptoms resolved, and the PSA level decreased slightly.

**Figure 5.**
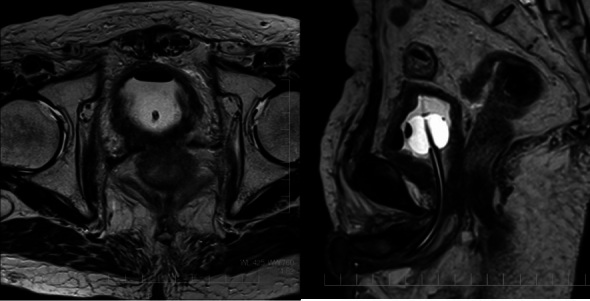
Pelvic MRI with urinary catheter shows significant reduction of the multiple bladder invasions at 9 months after HDR-BT. HDR-BT, high-dose-rate brachytherapy.

At 9 months after HDR-BT, MRI revealed significant shrinkage of the multiple bladder invasions (Figure 5). The patient was unable to retain urine before or after radiotherapy, and his maximum bladder volume was approximately 50 ml. Therefore, we placed a urinary catheter to fill the bladder with saline during MRI examinations. Dysuria and hematuria resolved 1 year after HDR-BT. Late toxicities included Grade 1 urinary incontinence (which pre-existed and did not worsen after radiotherapy) and Grade 1 bloody stool. At 22 months after HDR-BT, the patient had Grade 1 urinary incontinence without hematuria and Grade 1 bloody stool. He continued receiving enzalutamide, but his PSA level continued to increase during follow-up. However, there was no urinary obstruction requiring nephrostomy or ureteral stent placement.

## Discussion

We achieved excellent local control of post-operative local recurrence of CRPC with multiple bulky bladder invasions using EBRT followed by an HDR-BT boost. In the present case, the CTV at the time of brachytherapy was 58.35 cc, indicating a very bulky tumor. To the best of our knowledge, the present report is the first to demonstrate the excellent local control of bulky local recurrence of prostate cancer.

Most adjuvant and salvage radiotherapies use EBRT alone because the target is unclear. Dose escalation protocols for EBRT with IMRT have achieved better tumor control rates.^6,7^ However, these treatments have been associated with higher grade GU toxicities such as bladder neck and vesicourethral anastomosis.

HDR-BT can achieve dose escalation for the gross target while limiting toxicities in the adjacent organs, such as the urethra and rectum, within tolerable levels.^8^

Radiobiologically, prostate cancer has a low α/β ratio; hence, hypofractionation schedules such as HDR-BT have a significantly greater biological effect than EBRT.^9^ The ASCENDE-RT trial showed that the rate of biochemical-progression-free survival was significantly higher with low-dose-rate prostate brachytherapy boost than that with 78 Gy EBRT.^10^ Approximately, 70% of the patients included in this trial had NCCN high-risk prostate cancer. Although no high-level randomized trial has compared HDR boost with EBRT alone for salvage treatment in local recurrence after prostatectomy, we hypothesized the same scenario as the ACENDE-RT trial.

Recent advances in imaging methods, such as multiparametric MRI and choline PET/CT, enable the detection of local and distant recurrences.^11^ More recently, prostate-specific membrane antigen PET-CT has emerged as a new, standard imaging modality, not only for determining the recurrence status, but also for the initial treatment of prostate cancer.^12–14^ Owing to these recent advances, local recurrence of prostate cancer after initial treatment can be detected at higher frequencies.

There are very few reports of salvage HDR-BT for recurrent prostate cancer after radical prostatectomy. Niehoff et al. treated 35 patients with TRUS-detectable recurrent tumors after radical prostatectomy using HDR-BT combined with EBRT (3D-CRT technique).^15^ They administered a BT boost (30 Gy in 2 fractions) after complementary EBRT in which 21 and 14 patients received a dose of 30 Gy and 40 Gy, respectively. This report did not indicate the CTV. After a mean follow-up of 27 months, 67% of the patients had elevated PSA levels with or without local recurrence and/or systemic progress. The mean duration of absence of biochemical evidence of disease was 12 months. There was no significant difference between patients who received 30 Gy EBRT and those who received 40 Gy EBRT. The study also found no acute or late Grade III/IV toxicity in any of the patients (LENTSOMA, RTOG/EORTC).

Strom et al. reported six patients with biopsy-proven recurrent prostate cancer after definitive prostatectomy treated with or without IMRT.^16^ Five patients received IMRT at a dose of 45–50.4 Gy in 25–28 fractions to the prostate bed followed by HDR-BT (19 Gy/2 fractions). The sixth patient received HDR-BT monotherapy at a dose of 38 Gy in four fractions over 3 days. The median CTV was 2.3 cc (range: 1.6–4.7 cc). The median follow-up period was 9 months, and at the last follow-up, all patients had undetectable PSA levels. One patient experienced late Grade 2 urinary incontinence. There were no cases of late gastrointestinal toxicity ≥Grade 2.

Buchser et al. reported 11 patients who received salvage HDR-BT (15 Gy/1 fraction) with EBRT (37.5 Gy/15 fractions) for histologically confirmed, locally relapsed macroscopic prostate cancer after radical prostatectomy with a median volume of 3.34 cc (range: 1.98–6.76 cc).^17^ At a median follow-up of 7 months, all patients showed an appropriate biochemical response, and the acute GU/gastrointestinal toxicity levels were acceptable; there were no cases of late toxicity.

Compared to the outcomes in the abovementioned studies, in the present case we achieved excellent local control of a very bulky tumor involving the bladder (CTV: 58.35 cc) using EBRT plus HDR-BT boost. EBRT plus HDR-BT boost allows higher doses to be administered than EBRT alone, and is associated with less toxicity even when treating bulky, locally recurrent prostate cancer following prostatectomy. Unfortunately, our patient developed multiple bone metastases 4 months after radiotherapy. Although we performed an FDG-PET scan to exclude distant metastasis at the time of initial radiotherapy, there may have been micrometastatic lesions before radiotherapy. As a result, our treatment may have been palliative, but not definitive. Recently, the STAMPEDE trial showed that palliative radiotherapy for the primary site had a significant benefit for overall survival in patients with prostate cancer with low metastatic burden.^18^ Therefore, even after palliative treatment, strong local control to prevent urinary obstruction is important in terms of patients’ quality of life.

Urinary obstruction is a major complication of advanced prostate cancer. Treatment for malignant ureteric obstructions includes percutaneous nephrostomy, ureteric stent insertion, or occasionally, other forms of urinary diversion. New et al. reviewed 184 patients who underwent percutaneous nephrostomy due to prostate cancer progression.^19^ They reported a survival after percutaneous nephrostomy of 4–31 months, with longer survival typically seen in patients who were hormone naïve or those who experienced good recovery of their renal function. Percutaneous nephrostomy is very effective for treating malignant urinary obstructions, but procedure-related complications necessitate frequent readmission. Nephrostomy may also influence patients’ physical activity levels and restrict their social lives.^20^ Our patient had Grade 1 urinary incontinence requiring the use of incontinence pads following radiotherapy; however, following salvage radiotherapy, the expected typical symptoms of urinary obstruction did not develop.

Our study has some limitations. First, there was a lack of initial perioperative findings. Second, although we used TRUS-, CT-, and MRI-guided brachytherapy to increase the accuracy of the needle insertions, the possibility of needle movement resulting in a slightly deeper insertion or needle removal during irradiation cannot be denied. Third, although EBRT plus HDR-BT boost is a potent local treatment, its long-term and late toxicities are unknown. Therefore, a long-term follow-up is required. At 22 months after HDR-BT, our patient had Grade 1 urinary incontinence and Grade 1 tarry stools. Currently, the patient is without any treatment and has not needed readmission due to late toxicity of radiotherapy, and is being continuously followed-up.

We achieved excellent local control of cancer in the prostate bed and multiple bulky bladder invasions. EBRT plus HDR-BT boost can allow higher doses to be delivered than EBRT alone for locally recurrent bulky prostate cancer following prostatectomy. However, treatment toxicity and indication of EBRT + HDR-BT boost should be thoroughly discussed considered among radiation oncology experts.

## Learning points

- EBRT followed by HDR-BT boost can enable control of bulky recurrence in the prostate bed and CRPC bladder invasion after prostatectomy.
- If the treatment is palliative, local control to prevent urinary obstruction is important in terms of the patient’s quality of life.
